# Optimization Study of Small-Scale Solar Membrane Distillation Desalination Systems (s-SMDDS)

**DOI:** 10.3390/ijerph111112064

**Published:** 2014-11-24

**Authors:** Hsuan Chang, Cheng-Liang Chang, Chen-Yu Hung, Tung-Wen Cheng, Chii-Dong Ho

**Affiliations:** Department of Chemical and Materials Engineering, Tamkang University, New Taipei City 25137, Taiwan; E-Mails: chlchang@mail.tku.edu.tw (C.-L.C.); tony94m6@gmail.com (C.-Y. H.); twcheng@mail.tku.edu.tw (T.-W.C.); cdho@mail.tku.edu.tw (C.-D.H.)

**Keywords:** solar energy, desalination, membrane distillation, optimization, dynamic modeling

## Abstract

Membrane distillation (MD), which can utilize low-grade thermal energy, has been extensively studied for desalination. By incorporating solar thermal energy, the solar membrane distillation desalination system (SMDDS) is a potential technology for resolving energy and water resource problems. Small-scale SMDDS (s-SMDDS) is an attractive and viable option for the production of fresh water for small communities in remote arid areas. The minimum cost design and operation of s-SMDDS are determined by a systematic method, which involves a pseudo-steady-state approach for equipment sizing and dynamic optimization using overall system mathematical models. Two s-SMDDS employing an air gap membrane distillation module with membrane areas of 11.5 m^2^ and 23 m^2^ are analyzed. The lowest water production costs are $5.92/m^3^ and $5.16/m^3^ for water production rates of 500 kg/day and 1000 kg/day, respectively. For these two optimal cases, the performance ratios are 0.85 and 0.91; the recovery ratios are 4.07% and 4.57%. The effect of membrane characteristics on the production cost is investigated. For the commercial membrane employed in this study, the increase of the membrane mass transfer coefficient up to two times is beneficial for cost reduction.

## 1. Introduction

Membrane distillation (MD) is a thermally-driven membrane separation process, in which only vapor molecules are transported through porous hydrophobic membranes. The driving force is the vapor pressure difference between the hot liquid feed side and the cold permeate side of the membrane. The latest comprehensive reviews of various aspects of MD technology, including the fundamental concept, membrane configuration, membrane characteristics, membrane modules, applications, heat and mass transfer mechanisms, thermal efficiency and energy consumption, fouling, as well as the effects of operating parameters, are by Alkhudhiri *et al.* [1] and Camacho *et al.* [2]. MD has many applications, such as desalination, heavy metal removal from waste water and aqueous solution concentration in the food industry. Desalination is the most studied MD application. The advantages of MD over other desalination processes include less sensitivity to feed concentration, the ability to use low temperature heat, the ability to use relatively cheap and robust membranes, high product quality, high system compactness and high fouling resistance [2].

Being capable of directly utilizing renewable solar thermal energy, the solar membrane distillation desalination system (SMDDS) has evolved as a promising technology for alleviating energy and water resource problems simultaneously. Small-scale SMDDS (s-SMDDS) is an attractive and viable option for the production of fresh water for small communities in remote arid areas. The EU-funded SMADES project (PV and thermally driven small-scale, stand-alone desalination systems with very low maintenance needs) [3] and MEDESOL project (seawater desalination by innovative solar-powered membrane distillation system) [4] have both developed and investigated s-SMDDS.

Qtaishat and Banat [5] reviewed the research efforts of coupling MD modules with various solar energy systems, including flat plate collectors, vacuum collectors, solar ponds, solar stills and parabolic troughs. The MD modules employed for SMDDS include hollow fiber modules, spiral-wound modules with heat recovery and compact flat plate modules. The MD configurations adopted for SMDDS include direct contact (DCMD), air gap (AGMD), liquid gap (LGMD) and vacuum (VMD) types. The small and lab-scale SMDDSs tested have shown that the MD process is suitable to operate in conjunction with solar energy for small capacities [5]. The few economic studies showed that the pure water production costs of SMDDS are much higher than other desalination technologies. Banat and Jwaied [6] estimated the costs of two s-SMDDS, which employ spiral-wound LGMD modules, developed in the SMADES project, to be $15/m^3^ and $18/m^3^ for a compact unit (specified by a 100-L/day capacity and a 10-m^2^ membrane area) and a large unit (specified by a 500-L/day capacity and a 40-m^2^ membrane area), respectively. In the MEDESOL project, the water production costs of three small stand-alone solar systems of different heat recovery configurations were analyzed [7]. The systems employed a flat plate AGMD module (of a 2.8-m^2^ membrane area), developed and manufactured by the Swedish company, Scarab AB. With specified operation conditions and solar collector area, the production costs estimated are $15.67/m^3^ for brackish water and $31.34/m^3^ for sea water. Recently, Saffarini *et al.* [8] evaluated the water production costs of three solar-powered MD desalination systems that employ DCMD, AGMD and VMD configurations, but with the same specified membrane area of 7 m^2^ and recovery ratio of 4.4%. The water production costs of the systems using DCMD, AGMD and VMD modules are $12.7/m^3^, $18.26/m^3^ and $16.02/m^3^, respectively. For the system using the AGMD module, Saffarini *et al.* [8] also examined the effects of design and operation parameters and concluded that these parameters can significantly affect the water production cost.

Although not specifically commenting on SMDDS, Khayet and Matsuura [9] pointed out that the commercial application of MD technology is hindered by energy efficiency and economics. Summers *et al.* [10] emphasized that most research on MD has focused on maximizing membrane flux as opposed to minimizing energy consumption and cost. However, in MD systems, membrane flux is not only determined by the membrane characteristics, but also highly dependent on the system configuration, membrane area, energy input and heat recovery from the hot fluid and condensing vapor. Furthermore, in a complete system, the highest membrane flux operation may not lead to the best use of energy or the lowest cost. It is imperative that minimum-cost SMDDS designs, which should be obtained via overall system optimization, be identified to justify the economic feasibility of SMDDS. In addition, the significance of enhancing membrane characteristics, which is the focus of much research, should be examined from the overall system cost reduction point of view.

The aim of this study is to determine the minimum water production cost of two s-SMDDS employing AGMD modules by a systematic method. In this paper, rigorous mathematical models for the equipment of the system, including the solar collector, thermal storage tank, heat exchanger and air gap membrane distillation module, are developed and integrated for the simulation of the overall system. The design and operation conditions of the s-SMDDS are then determined via dynamic optimization. The equipment sizes of the s-SMDDS, which are operated with unsteady solar radiation, are determined by a pseudo-steady-state approach. With the simulation models, the effect of membrane characteristics on the water production cost is analyzed by varying the mass transfer resistance of the membrane.

## 2. System and Modeling

**Figure 1:**
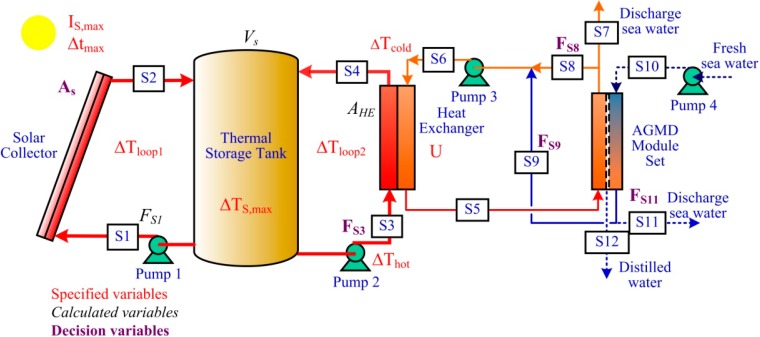
The small-scale solar membrane distillation desalination system (s-SMDDS). AGMD, air gap membrane distillation.

In this study, a flowsheet of the s-SMDDS using AGMD modules, as depicted in Figure 1, is proposed. The system includes a solar collector, a thermal storage tank, a heat exchanger, an AGMD module and four pumps. Instead of PV (photovoltaic) modules, the electricity needed comes from an electric grid. The flowsheet includes two closed circulation loops, *i.e.*, the solar collector-thermal storage tank loop (Loop 1) and the thermal storage tank-heat exchanger loop (Loop 2). For the AGMD module, both hot side and cold side heat recovery configurations are included in the flowsheet. In the hot side recovery configuration, the hot side outlet stream (S8) of the MD module is sent to the heat exchanger for further heating. In the cold side configuration, the cold side outlet stream (S9) of the MD module is sent to the heat exchanger for further heating. These two heat recovery configurations have been proposed and analyzed in [7].

As with other thermal and chemical processes, the individual equipment of SMDDS, such as the MD modules, can be simulated by developing models from fundamental principles. Furthermore, one can build the models on many commercial process simulation platforms, which enable the easy study of the design alternatives of the equipment and the overall flowsheet. Chang *et al.* have reported the model development and the flowsheet analysis for the SMDDS using DCMD and AGMD modules on Aspen Plus^®^ and Aspen Custom Modeler^®^ platforms [11,12,13,14].

One-dimensional (1D) models are developed for individual equipment. Considering the differences in time constants of the equipment, not all of the dynamics of the equipment are included in the models. For the solar collector, thermal storage tank and heat exchanger, only the thermal dynamics are considered. For the MD module, the transients of both mass flow and energy flow are ignored.

For the solar collector, the energy balance taking into account the energy flows associated with mass convection and solar radiation with the collector efficiency (η_sc_) is:

(1) $$\frac{\partial T_{f,sc}}{\partial t}=-L_{SC}\frac{m_{f,sc}}{M_{f,sc}}\frac{\partial T_{f,sc}}{\partial x}+\frac{A_{sc}I(t)\eta_{sc}}{M_{f,sc}C_{p}}$$

For the thermal storage tank, because the size is small for the s-SMDDS, the ideal temperature stratification inside the tank may not be achievable. A conservative approach is taken in this study. The hot and cold inlet streams enter the tank concurrently. The temperature variation in the thermal storage tank with a circulation flow rate of m_f,ST_ and the inlet stream, which is the combination of S2 and S4, can be determined from the energy balance as:

(2) $$\frac{\partial T_{f,ST}}{\partial t}=-H_{ST}\frac{m_{f,ST}}{M_{f,ST}}\frac{\partial T_{f,ST}}{\partial x}$$

In the counter-current flow heat exchanger, the hot fluid comes from the thermal storage tank and the cold fluid comes from the MD module. The energy balances for both fluids, considering the energy flow of mass convection and the heat transfer between both fluids, are given as:

(3) $$\frac{dT_{f,HX,HL}}{dt}=L_{HX}\frac{m_{f,HX,HL}}{M_{f,HX,HL}}\frac{\partial T_{f,HX,HL}}{\partial x}-\frac{A_{HX}U_{HX}}{M_{f,HX,HL}C_{p}}(T_{f,HX,HL}-T_{f,HX,CL})$$

(4) $$\frac{dT_{f,HX,CL}}{dt}=L_{HX}\frac{m_{f,HX,CL}}{M_{f,HX,CL}}\frac{\partial T_{f,HX,CL}}{\partial x}-\frac{A_{HX}U_{HX}}{M_{f,HX,CL}C_{p}}(T_{f,HX,HL}-T_{f,HX,CL})$$

**Figure 2:**
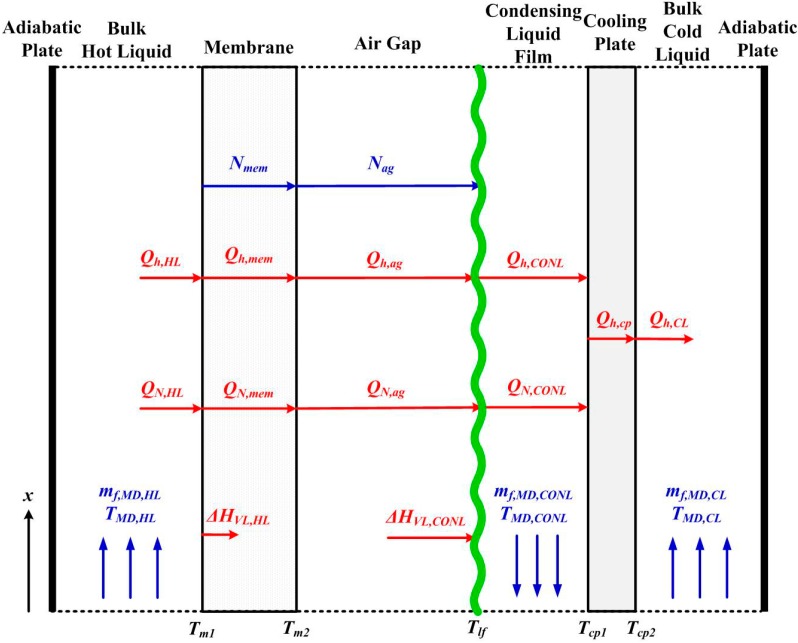
Heat and mass transfer mechanisms of AGMD.

For the AGMD module, a steady-state 1D model considering the heat and mass transfers in each layer and at the interface between layers, as illustrated in Figure 2, is developed. Mass balance equations can be written for the hot fluid and the condensing liquid, as well as for the interface between membrane and air gap as:

(5) $$\frac{dm_{f,MD,HL}}{dx}=-N_{mem}Mw_{w}L_{MD}$$

(6) $$\frac{dm_{f,MD,CONL}}{dx}=-N_{ag}Mw_{w}L_{MD}$$

(7) $$N_{mem}=N_{ag}$$

The mass fluxes are determined by the effective mass transfer coefficients and the pressure difference driving forces in the membrane and air gap layers. For the mass transfer in the membrane, Knudsen diffusion and molecular diffusion are taken into account [15]. For the air gap, only molecular diffusion is considered.

(8) $$N_{mem}=\frac{k_{mem}}{R{\bar{T}}_{mem}}(P_{sat,w,m1}-P_{w,m2})$$

(9) $$N_{ag}=\frac{k_{ag}P_{ag}^{T}}{R{\bar{T}}_{ag}P_{a,lm}}(P_{w,m2}-P_{sat,w,lf})$$

(10) Kmem=ετ[11Dk+yair,lmDm]1δmem

(11) $$h_{mem}=\frac{\varepsilon K_{V}+K_{mem}(1-\varepsilon )}{\delta_{mem}}$$

The energy balances for the hot and cold fluid channels, in addition to the energy flow of mass convection, the convective heat transfer and the sensible heat effect associated with the mass transfer across the boundaries are taken into account.

(12) $$\frac{\partial T_{f,MD,HL}}{\partial t}=-L_{MD}\left[\frac{m_{f,MD,HL}}{M_{f,MD,HL}}\frac{\partial T_{f,MD,HL}}{\partial x}+\frac{W_{mem}}{M_{f,MD,HL}C_{p}}\left(Q_{h,HL}+Q_{N,HL}\right)\right]$$

(13) $$\frac{\partial T_{f,MD,CL}}{\partial t}=-L_{MD}\left[\frac{m_{f,MD,CL}}{M_{f,MD,CL}}\frac{\partial T_{f,MD,CL}}{\partial x}-\frac{W_{mem}}{M_{f,MD,CL}C_{p}}Q_{h,CL}\right]$$

For each interface, the heat effects on both sides should be balanced.

(14) $$Q_{h,HL}+Q_{N,HL}-\Delta H_{VL,HL}=Q_{h,mem}+Q_{N,mem}=Q_{h,ag}+Q_{N,ag}=Q_{h,CONL}+Q_{N,CONL}-\Delta H_{VL,CONL}=Q_{h,cp}-\Delta H_{VL,CONL}=Q_{h,CL}-\Delta H_{VL,CONL}$$

The heat fluxes of convective heat transfer, sensible heat transfer and latent heat transfer are determined by:

(15) $$Q_{h}=h\Delta T$$

(16) $$Q_{N}=NC_{p}\Delta T$$

(17) $$\Delta H_{VL}=N\Delta H_{vap}$$

The heat transfer coefficients for hot fluid and cold fluid channels are estimated using the correlation for laminar flow and constant wall heat flux [16].

(18) Nu=4.36+0.036[RePrLDh]1+0.011[RePrLDh]0.8

For the liquid film, the heat transfer coefficient is determined using the correlation for condensing film [16].

(19) $$h_{CONL}=0.943\left[\frac{\rho_{CONL}\left(\rho_{CONL}-\rho_{V}\right)\Delta H_{vap}K_{CONL}}{L\mu_{CONL}\left(T_{CONL}-T_{mp}\right)}\right]$$

For the membrane, air gap and cooling plate, the heat transfer coefficients are determined using the thermal conductivity and thickness of the layer. The membrane thermal conductivity is determined from the thermal conductivities of the solid membrane and vapor inside the pore by using membrane porosity (ε) as the weighting factor.

(20) $$h_{ag}=\frac{K_{ag}}{\delta_{ag}}$$

(21) $$h_{cp}=\frac{K_{cp}}{\delta_{cp}}$$

The models are built on the commercial Aspen Custom Modeler^®^ platform and solved using the built-in solver. The partial differential equations are transformed into differential algebraic equations using the method of lines first and then solved by Newton’s method [17]. In the previous studies [13,14], the models were verified with a laboratory flat plate AGMD module and a laboratory-scale simulated SMDDS.

## 3. Equipment Sizing

**Figure 3:**
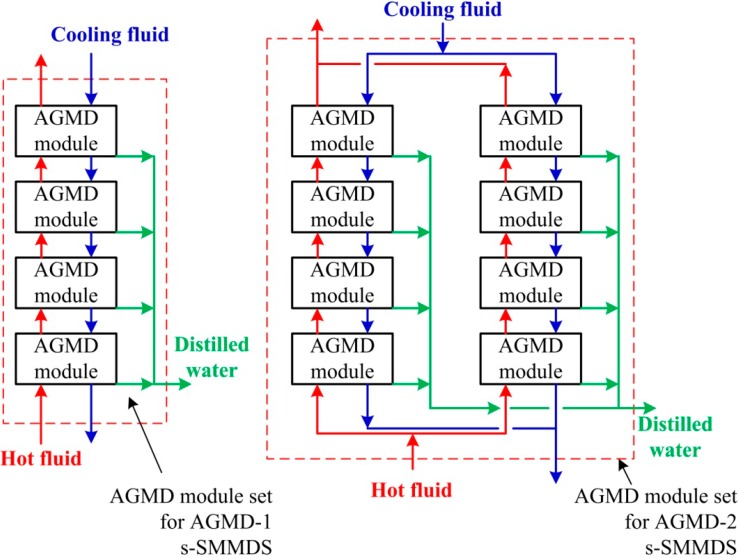
AGMD module arrangements for AGMD-1 and AGMD-2.

AGMD modules using feed channel spacers to improve performance have been proposed and evaluated in the literature [18,19]. For the purpose of this study, commercial flat sheet simple-channel modules are adopted. More specifically, the dimensions of the AGMD modules are defined to be the same as that of the flat sheet commercial module developed and manufactured by the Swedish company, Scarab AB. The Scarab module has been adopted in the solar desalination pilot plant of the MEDESOL project [20]. Each module consists of 10 plastic cassettes with a total membrane area of 2.88 m^2^. The attributes of the AGMD module are summarized in Table 1. The two s-SMDDS analyzed are named the AGMD-1 and AGMD-2 systems. For the AGMD-1 system, the four modules are connected in series and the total membrane area is 11.5 m^2^. For the AGMD-2 system, two sets of four-in-series are connected in parallel and the total membrane area is 23 m^2^. The arrangements of these two systems are depicted in Figure 3.

**Table 1:** Attributions of the AGMD module.

Parameter	Value
Total membrane area (m^2^)	2.8
Single sheet membrane width (m)	0.36
Single sheet membrane length (m)	0.39
Membrane material (porous + supporting)	PTFE + PP
Membrane thickness (μm)	30/170
Membrane pore diameter (μm)	0.2
Membrane porosity	0.8
Height of hot fluid channel (mm)	1
Height of cold fluid channel (mm)	1
Thickness of air gap (mm)	1

Because of the intermittent and dynamic nature of solar radiation, s-SMDDS is not operated under steady states. A pseudo-steady-state approach is proposed to determine the sizes of three major pieces of equipment, including the thermal storage tank, the heat exchanger and the circulation pump of Loop 1. The concept is to view the s-SMDDS as a system operated at a pseudo-steady state to transfer an amount of solar heat throughout the process, *i.e.*, from the solar collector end to the MD unit end. The sizes of the equipment can then be determined based on the heat transfer rate and several specified variables, which are marked in red in Figure 1 and discussed in the following.

The size of the thermal storage tank (V_ST_) is determined by specifying the maximum temperature rise of the water in the tank over the maximum solar heat input period. For the solar collector with a specified area (A_SC_) operated under the maximum solar radiation intensity (I_Smax_) and the collector efficiency (η_SC_), the maximum heat transfer rate (q_PSmax_) from the solar collector to the thermal storage tank can be determined by:

(22) $$q_{PS\text{max}}=A_{SC}I_{S\text{max}}\eta_{SC}$$

**Figure 4:**
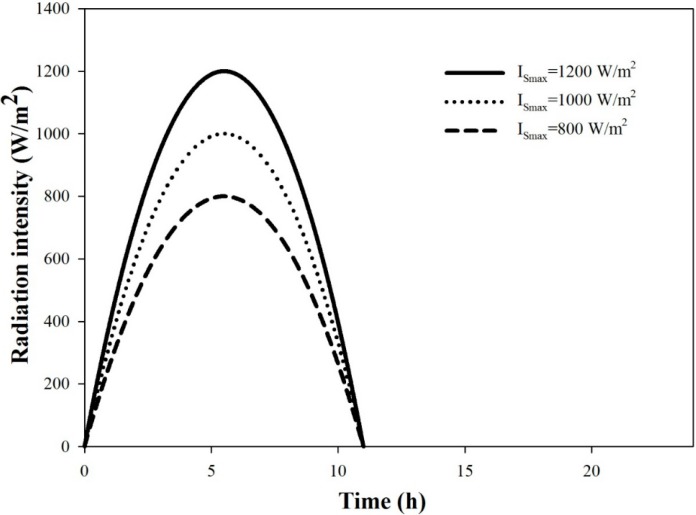
Solar radiation profiles.

In this study, the solar radiation profiles used, as shown in Figure 4, are parabolic with different specified maximum intensity, but the same day-time period of 11 h. The solar collector efficiency is assumed to be 50% [8].

The energy balance for the water in the thermal storage tank over a time period of (Δt_max_) and a specified maximum temperature rise (ΔT_Smax_) of the water in the tank is given as:

(23) $$q_{PS\text{max}}\Delta t_{\text{max}}=\rho V_{ST}C_{p}\left(\Delta T_{S\text{max}}\right)$$

The size of the circulation pump of Loop 1 (F_S1_) is determined using the maximum heat transfer rate and a specified temperature rise of Loop 1 (ΔT_Loop1_) by:

(24) $$q_{PS\text{max}}=F_{S1}C_{p}\Delta T_{Loop1}$$

The size of the heat exchanger (A_HX_) is determined by the pseudo-steady heat transfer rate (q_PS_) in the heat exchanger with a specified logarithm mean temperature difference (ΔT_lm_) and an assumed overall heat transfer coefficient (U_HX_). Given the specified circulation flow rate of S3 (F_S3_) and the temperature rise of Loop 2 (ΔT_Loop2_), the heat transfer rate is calculated by:

(25) $$q_{PS}=F_{S3}C_{p}\Delta T_{Loop2}$$

The area of the heat exchanger can then be calculated from the following design equation:

(26) $$q_{PS}=U_{HX}A_{HX}\Delta T_{lm}$$

The sizes of equipment other than the thermal storage tank, Pump 1 and heat exchanger are determined by dynamic optimization, including the solar collector, Pump 2, Pump 3 and Pump 4. In other words, they are the decision variables in the optimization problem.

## 4. Cost Analysis

The capital, operating and total annual costs of the s-SMDDS are analyzed according to the following bases adopted by Banat and Jwaied [6]:

- The installation cost is 25% of the purchased equipment costs.
- The instrumentation and control cost is 25% of the total purchased equipment cost.
- Zero land cost.
- Zero pretreatment cost.
- The annual interest rate and plant lifetime for amortization of the capital cost or determining the annual fixed charge are 5% and 20 years.
- The annual operating and maintenance (O&M) cost is estimated to be 20% of the plant annual fixed charge.
- The membrane replacement rate is 20% per year.

The equipment costs are determined using the cost functions listed in Table 2. The cost function of the solar collector with a rack is based on the costs reported in [6] and has been adjusted with the CEPCI (Chemical Engineering Plant Cost Index) of 2013. The unit cost of the membrane module is based on that used in [6,7] and is also adjusted with 2013 CEPCI. The cost functions of the thermal storage tank, heat exchanger and pump are developed by this study based on the information provided by the suppliers. All of the pumps are specified to provide a water head of 20 m. For the heat exchanger and the pumps for brackish or sea water, a material capital cost factor (F_M_) is applied to account for the anti-corrosion material used for the construction.

**Table 2:** Cost functions for equipment.

Equipment	Purchased Cost ($)	Notes
Solar collector	CSC=890.78(ASC5.73)0.9	With rack.
Thermal storage tank	CS=165(VS1000)0.57	Carbon steel.
Plate heat exchanger	$C_{HE}=\left[363.56+8.54\left(\frac{A_{HE}-1}{0.032}\right)\right]F_{M}$	$F_{M}=3.5$ for anti-corrosion material of construction; 1 m^2^ ≤ A_HE_ ≤ 5 m^2^
Pump	Cp=265.4(F95)0.4FM	$F_{M}=3.5$ for anti-corrosion material of construction;
		$F_{M}=1$ for carbon steel;
		For a pumping head of 20 m.
Membrane module	$C_{MD}=410A_{mem}$	Flat sheet AGMD membrane module as the product based on [7] and modified cost index;
		PTFE membrane.

The unit cost of water production (c_w_) is obtained from the total annual cost (TAC) of the system and the daily water production rate (F_DW_), as given by:

(27) $$c_{w}=\frac{TAC}{F_{DW}\times 365}$$

The total annual cost is the sum of annual fixed charge (C_fixed_), membrane replacement cost (C_mr_), O&M (C_O&M_) and electricity costs (C_elec_).

(28) $$TAC=C_{fixed}+C_{mr}+C_{O\&M}+C_{elec}$$

The annual fixed charge can be calculated from the total purchased cost of all equipment (C_CC_) with the 25% installation cost and the amortization factor (a) as:

(29) $$C_{fixed}=a\left(1+25\%\right)C_{CC}$$

With the annual interest rate (i) and plant lifetime (n), the amortization factor is determined by:

(30) $$a=\frac{i{\left(1+i\right)}^{n}}{{\left(1+i\right)}^{n}-1}$$

## 5. Dynamic Optimization

Using the dynamic models of the s-SMDDS, the design of the system that leads to the lowest unit cost for a specified water production rate can be found. The optimization problem is defined as:

(31) $$\min(c_{w})=f\left(A_{SC},F_{S3},F_{S8},F_{S9},F_{S11}\right)$$

subject to:

- the desired distilled water production rate (F_DW_);
- the solar radiation profile (I_S_);
- the parameters for pseudo-steady-state analysis; ΔT_Smax_, ΔT_lm_, ΔT_Loop1_, ΔT_Loop2_;
- the maximum temperature of S2 ( T_S2_ < 95 °C).

For each set of decision variables of the optimization problem, as specified in Equation (32), the sizes of the thermal storage tank, heat exchanger and Pump 1 are determined by the methods explained in Section 3. The flow rates of S3, S8, S9 and S11 are constant for the entire operation period. On the contrary, the flow rate of S1 is time dependent. It is determined by the instantaneous solar heat input, the target temperature of S2 (95 °C) and the temperature of S1:

(32) $$A_{SC}I_{S}\eta_{SC}=F_{S1}C_{p}\left(95-T_{S1}\right)$$

The system operation will be stopped when the temperature of thermal storage tank is lower than 50 °C.

For both AGMD-1 and AGMD-2 systems, this study implements the optimization analysis for different specified daily water production rates, respectively. The FEASOPT (feasible path successive quadratic programming) algorithm built-in in the Aspen Custom Modeler^®^ platform is adopted for the optimization search.

**Figure 5:**
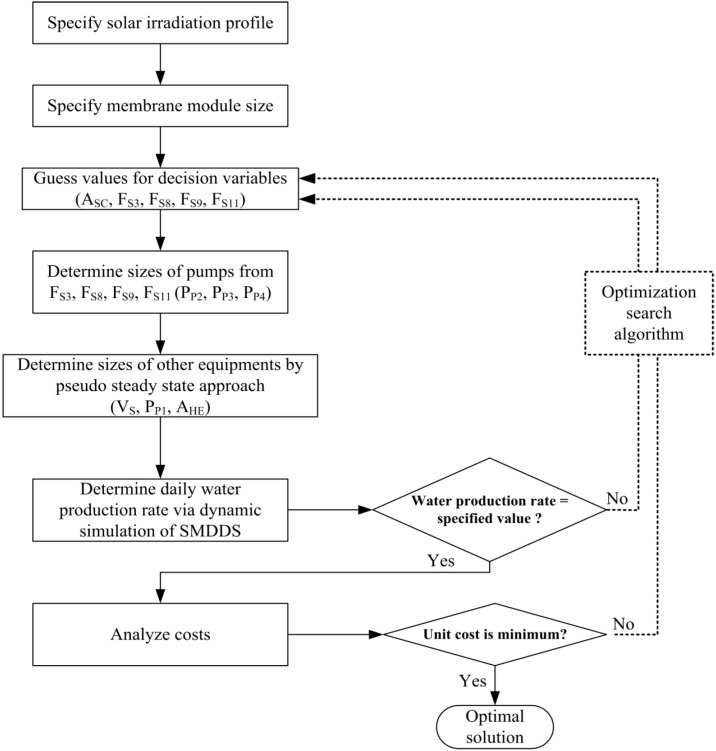
The systematic optimization method.

The systematic optimization method discussed above is summarized in Figure 5.

## 6. Results and Discussion

### 6.1. Optimal Solutions and Performance

Because of the high purity of the water produced, in the case where potable water is required, the pure water product can be blended with raw water in order to achieve adequate potable water. In the previous economic evaluation studies [6,7,8], the unit cost with a 1:1 dilution of the pure water produced is used for comparison with the production costs from other desalination technologies. In this study, the cost data presented are the 1:1 dilution costs; however, the water production rate refers to the pure water produced from s-SMDDS. The 1:1 dilution cost is only used for comparison with the literature data. It is not an assumption of the analysis and has no effects on the optimal design and cost results.

The minimum unit cost solutions for the AGMD-1 system, which uses 11.5 m^2^ of membrane area and is operated for a daily water production rate of 100–600 kg/day, are summarized in Table 3. The unit production costs ranged from $5.92/m^3^ to $15.7/m^3^. Both the equipment sizes and the circulation flow rates increase with the water production rate. In the optimization analysis, the minimum size of the heat exchanger is set as 1 m^2^. The lowest cost case for the AGMD-1 system is when 500 kg/day of pure water is produced with a solar collector area of 47.6 m^2^ and a heat exchanger area of 2.55 m^2^. The minimum unit cost solutions for the AGMD-2 system, which uses a 23-m^2^ membrane area, operated for a daily water production rate of 200–1000 kg/day, are summarized in Table 4. The unit production costs fall between $5.16/m^3^ and $14.24/m^3^, which are lower than those of the AGMD-1 system. The lowest cost case for the AGMD-2 system is when 1000 kg/day of pure water is produced with a solar collector area of 89.6 m^2^ and a heat exchanger area of 3.53 m^2^. The equipment sizes and costs of the lowest cost case of AGMD-1 system are compared with the reported data from [6,7,8] in Table 5. The unit cost of AGMD-1 is about 1/3 the literature reported data.

**Table 3:** Optimal solutions for the AGMD-1 system. STEC, specific thermal energy consumption; PR, performance ratio; RR, recovery ratio.

F_DW_ (kg/day)	100	200	300	400	500	600
Unit cost with 1:1 dilution ($/m^3^)	15.70	8.54	6.55	6.01	5.92	7.05
STEC (kWh/m^3^)	109.29	213.88	393.18	572.63	758.87	1424.76
PR	5.91	3.02	1.64	1.13	0.85	0.45
RR (%)	5.49	5.33	4.60	4.29	4.07	2.75
A_SC_ (m^2^)	1.36	5.35	14.76	28.70	47.57	107.21
V_ST_ (m^3^)	0.07	0.28	0.76	1.48	2.46	5.54
A_HX_ (m^2^)	1	1	1.01	1.66	2.55	2.97
F_S3_ (kg/h)	85.67	216.49	219.49	360.82	554.60	645.10
F_S8_ (kg/h)	0	0	0	0	0	0
F_S9_ (kg/h)	93.73	183.85	313.24	442.19	578.05	1015.95
F_S11_ (kg/h)	0	0	0	0	0	0

**Table 4:** Optimal solutions for the AGMD-2 system.

F_DW_ (kg/day)	200	400	600	800	1000
Unit cost with 1:1 dilution ($/m^3^)	14.24	7.84	5.95	5.23	5.16
STEC (kWh/m^3^)	100.88	242.76	371.43	533.41	713.76
PR	6.40	2.66	1.74	1.21	0.91
RR (%)	5.56	5.07	4.81	4.55	4.57
A_SC_ (m^2^)	2.52	12.17	27.95	53.54	89.58
V_ST_ (m^3^)	0.13	0.63	1.44	2.77	4.63
A_HX_ (m^2^)	1	1	2.77	4.60	3.53
F_S3_ (kg/h)	188.00	217.58	601.76	999.64	1536.21
F_S8_ (kg/h)	0	0	0	0	0
F_S9_ (kg/h)	180.95	399.48	591.36	824.45	1121.01
F_S11_ (kg/h)	0	0	0	0	0

**Table 5:** Comparison of equipment sizes and cost data for s-SMDDS.

Items	This study	Banat and Jwaied [6] (compact/large)	MEDESOL [7] ^2,3^	Saffarini *et al.* [8]
Capacity (kg/day)	500	100/500	73	700
Unit cost ^1^ ($/m^3^)	5.92	15/18	15.67	18.26
Equipment size				
*Membrane area (m^2^)*	11.5	10/40	2.3	7
*Solar collector area (m^2^)*	47.57	5.73/72	2.6	N/A
*Heat exchanger area (m^2^)*	2.55	0/N/A	$846	N/A
*Thermal storage tank (m^3^)*	2.46	N/A	N/A	N/A
Cost data				
*Membrane module*	$4730	$1080/$4320	$808 ($360/m^2^)	$350/m^2^
*Solar collector*	$5985 w/ rack	$900/$8700 w/ rack	$385 ($150/m^2^, w/o rack)	$160/m^2^ (w/o rack)
*Piping and tanks*	$275	$200/$500	$62	$250
*Heat exchanger*	$2730	0/$1500	$846	$750
*Pumps*	$1000	$300/$700	$150	$700
*Monitoring and control*	3680	$3328/$10,510	$385	$4500

Notes: **^1^** Unit cost with 1:1 dilution; **^2^** MEDESOL project (seawater desalination by innovative solar-powered membrane distillation system); **^3^** the IC4 case: heat recovery at the cold side, and the performance ratio is three.

For all of the optimal solutions of AGMD-1 and AGMD-2 systems, the flow rates of S8 and S11 are zero. These results reveal that: (1) the hot side recovery configuration is not beneficial to the overall system performance; and (2) the cold side recovery configuration should be operated by sending the entire cold side fluid out of the MD to the heat exchanger, *i.e.*, without partial discharge. The MEDESOL project [7] also concluded that the cold side recovery configuration is better.

**Figure 6:**
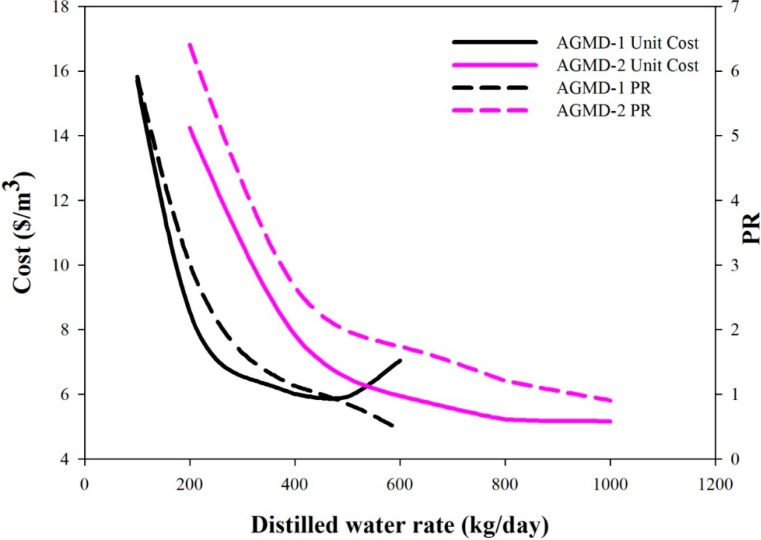
The effects of the water production rate on unit cost and PR.

**Figure 7:**
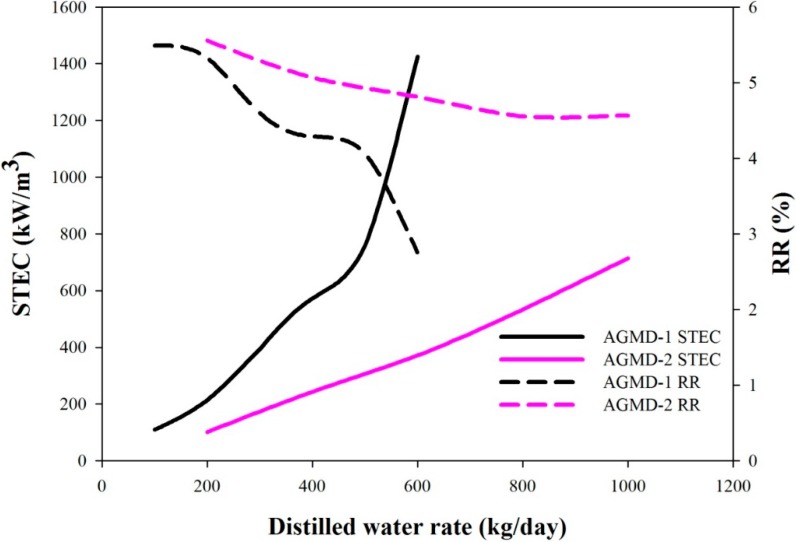
The effects of the water production rate on STEC and RR.

The desalination systems are commonly evaluated using several performance indexes, including PR (performance ratio, kg of water produced by the thermal energy of 1 kg steam), STEC (specific thermal energy consumption, kWh/m^3^) and RR (recovery ratio; ratio of the distillate rate to feed rate). For both AGMD-1 and AGMD-2 systems, the variations of these indexes with the water production rates are shown in Figure 6 and Figure 7. In Figure 6, PR decreases with the increase of the water production rate. In the same figure, the unit cost decreases with the increase of the water production rate up to 500 kg/day, but increases for higher water production rates. Optimal water production rates with the lowest unit costs can be determined. If the design criteria of pursuing higher PR were adopted, because no optimal values can be found, one will choose the designs with lower water production rates, but higher unit cost. The PR values corresponding to the lowest unit costs are 0.85 and 0.91 for the AGMD-1 system and the AGMD-2 system, respectively. Since STEC and PR are counter performance indexes, the remarks on the STEC curves shown in Figure 6 are similar to that of the PR results. The RR values decrease with the increase of water production rates, as shown in Figure 7. For the lowest unit cost cases of the AGMD-1 system and the AGMD-2 system, the RR values are 4.07% and 4.57%, respectively. In summary, the optimal solutions with the lowest unit costs do not correspond to operations with higher performance in terms of the common indexes of PR, STEC and RR.

The reasons that the unit costs of the optimal systems designed in this study are much lower than the literature reported costs are:

- For the systems reported in the literature, the sizes of equipment units and operating conditions are either not rigorously determined or are determined by a steady state analysis with a constant solar radiation intensity.
- The systems reported in this study are designed via dynamic optimization. For the fixed membrane module sizes, all other equipment units are optimally sized. The flow rates of all of the streams are also optimally determined, including the optimal time-varying flow rate of the solar collector circulation flow (S1). The flow rate of S1 varies with solar radiation and leads to the higher temperature of the hot fluid in the MD module through the heat transfer via the thermal storage tank and the heat exchanger.

### 6.2. Sensitivity of Pseudo-Steady-State Parameters

When employing the pseudo-steady-state approach, several parameters are specified, including ΔT_Smax_, ΔT_lm_, ΔT_Loop1_, and ΔT_Loop2_. The sensitivity of the values of these parameters should be examined. A set of base values of these parameters are specified as 10 °C, 5 °C, 20 °C and 25 °C for ΔT_Smax_, ΔT_lm_, ΔT_Loop1_ and ΔT_Loop2_, respectively. For the lowest unit cost case of the AGMD-1 system, *i.e.*, a production rate of 500 kg/day, the sensitivity analysis is conducted by varying the parameters one by one. The results are listed in Table 6. The effects of these parameters are not significant, except for very small ΔT_Smax_ and ΔT_Loop1_.

**Table 6:** Sensitivity analysis of the pseudo-steady-state parameters.

ΔT_Smax_ (°C)	5	10	15
Unit cost ($/m^3^)	7.74	**5.92**	5.12
ΔT_lm_ (°C)	**5**	10	15
Unit cost ($/m^3^)	**5.92**	5.77	5.71
ΔT_Loop1_ (°C)	15	**20**	25
Unit cost ($/m^3^)	N/A	**5.92**	5.91
ΔT_Loop2_ (°C)	20	**25**	30
Unit cost ($/m^3^)	5.82	**5.92**	6.03

Notes: 1. Unit cost is with a 1:1 dilution. 2. Bold face figures are base values. 3. N/A, the production rate of 500 kg/day cannot be obtained.

### 6.3. Operation Performance of Optimal Systems

**Figure 8:**
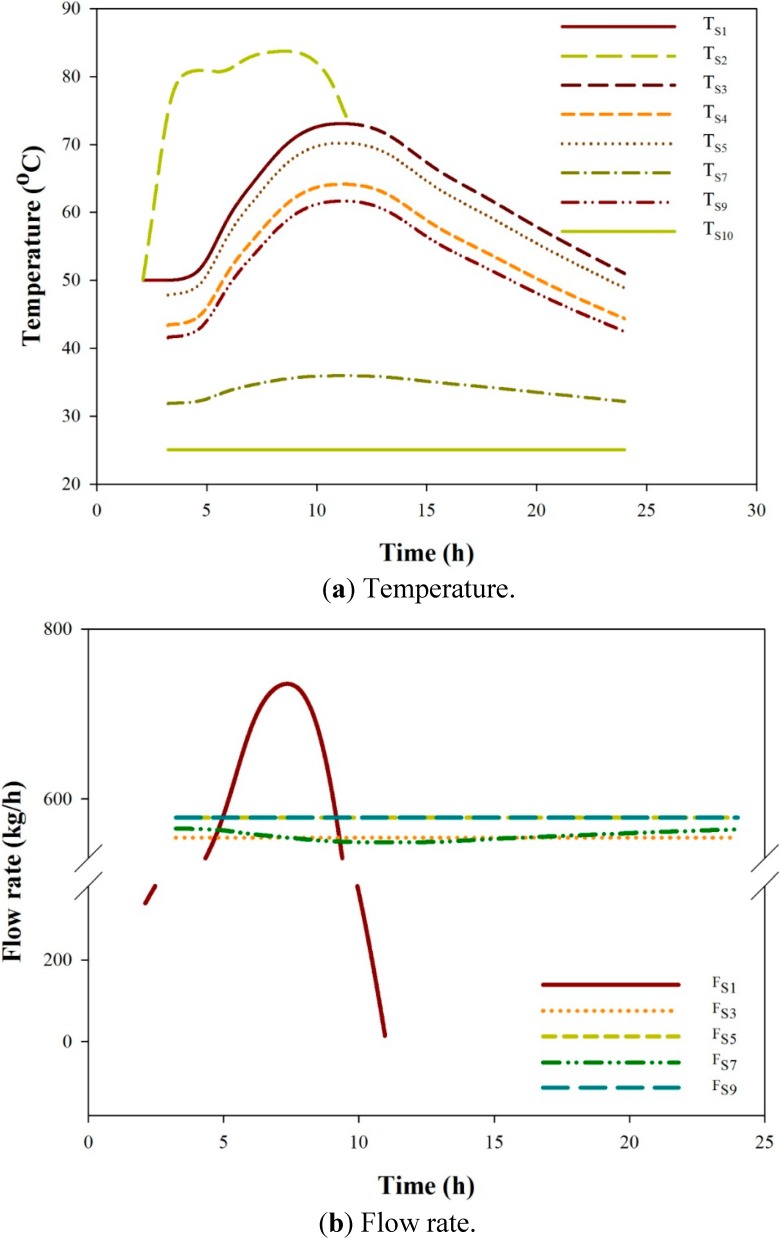

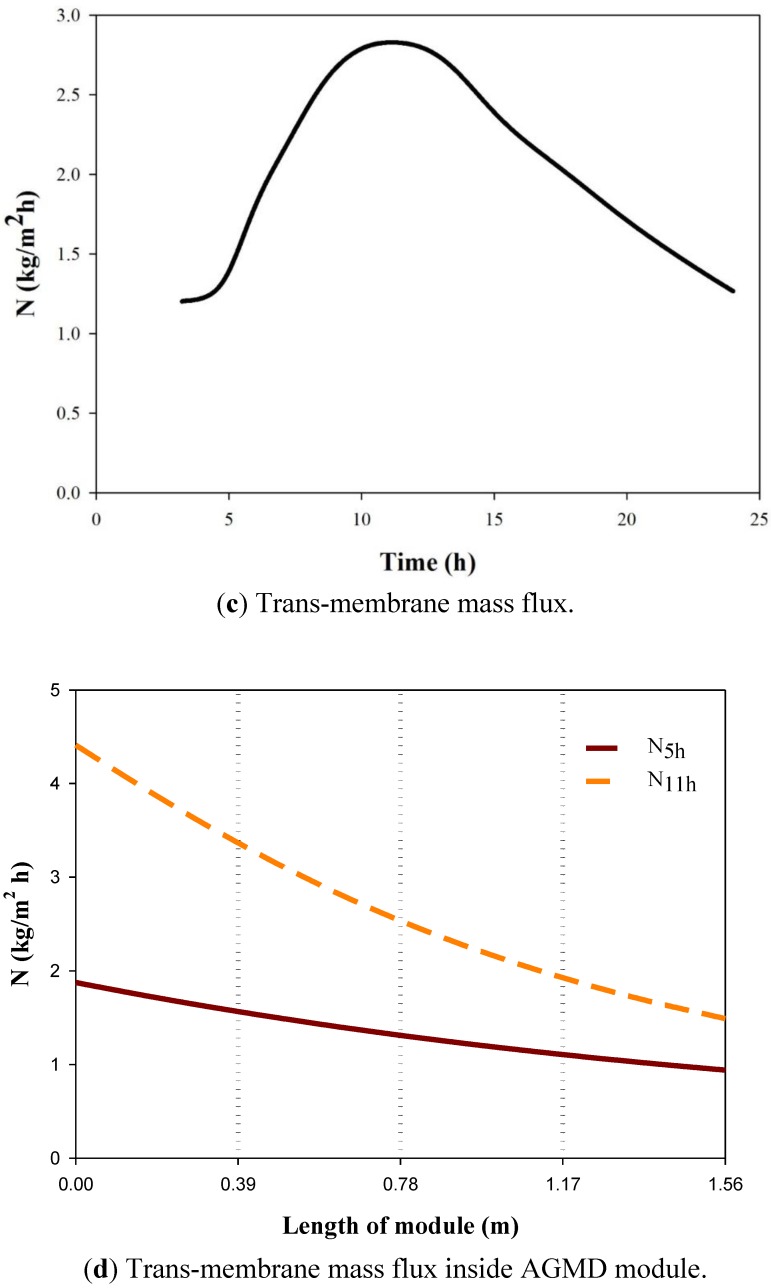
Optimal profiles of the AGMD-1-500 kg/day system for I_max_ = 1200 W/m^2^.

For the convenience of discussion, the optimal AGMD-1 system with the lowest unit cost, *i.e.*, a 500 kg/day production rate, is called the AGMD-1-500 kg/day system. When operated under the solar radiation profile with I_Smax_ equal to 1200 W/m^2^, the daily operation profiles of the AGMD-1-500 kg/day system are presented in Figure 8. The solar collector operates for 9.5 h, starting from the second hour after sunrise. Other units, including the thermal storage tank, heat exchanger and MD, operate for about 21 h, starting from the third hour after sunrise. The temperature profiles of all streams are shown in Figure 8a. The flow rate of Pump 1 is determined by Equation (25) and varies with time, which is different from other pumps with constant flow rates. The temperature profile of S2 is hence also different from that of those streams with constant flow rates. For all heated streams, the profiles are dome shaped, but the profile of S2 has a longer high temperature period. For all heated streams, except S2, the time when the maximum temperature occurs is delayed from the time at maximum solar radiation for about 5.5 h. The maximum temperature of S2 is 83.7 °C. The temperature differences between streams are greater when the stream temperatures are higher. The temperature of the discharged stream S7 falls between 31.8 °C and 35.9 °C. This indicates that the heat recovery in the MD module is effective.

The flow rates of major streams, including S1, S3, S5, S7 and S9, are shown in Figure 8b. Because S8 is zero, S5 and S9 have the same flow rates. The flow rate of S7 is lower than that of S5 by the amount of distilled water S12 and fluctuates because of the time-varying production rate. The flow rate of S1 is varied according to Equation (33) and has a maximum flow rate at about 1.8 h after the maximum radiation time.

The trans-membrane mass flux profile is shown in Figure 8c. The flux is between 1.2 and 2.8 kg/m^2^·h. The time of maximum flux corresponds to that of the highest stream temperature. The trans-membrane mass flux profiles inside the four AGMD modules connected in-series at five hours and 11 h after sunrise are shown in Figure 8d. At the time of five hours, when the system is operated at a lower temperature level, the mass fluxes decrease linearly from the hot fluid inlet to the outlet. At the time of 11 h, when the system is operated at the highest temperature level, the mass fluxes are significantly higher at the first two modules from the hot fluid inlet end.

**Figure 9:**
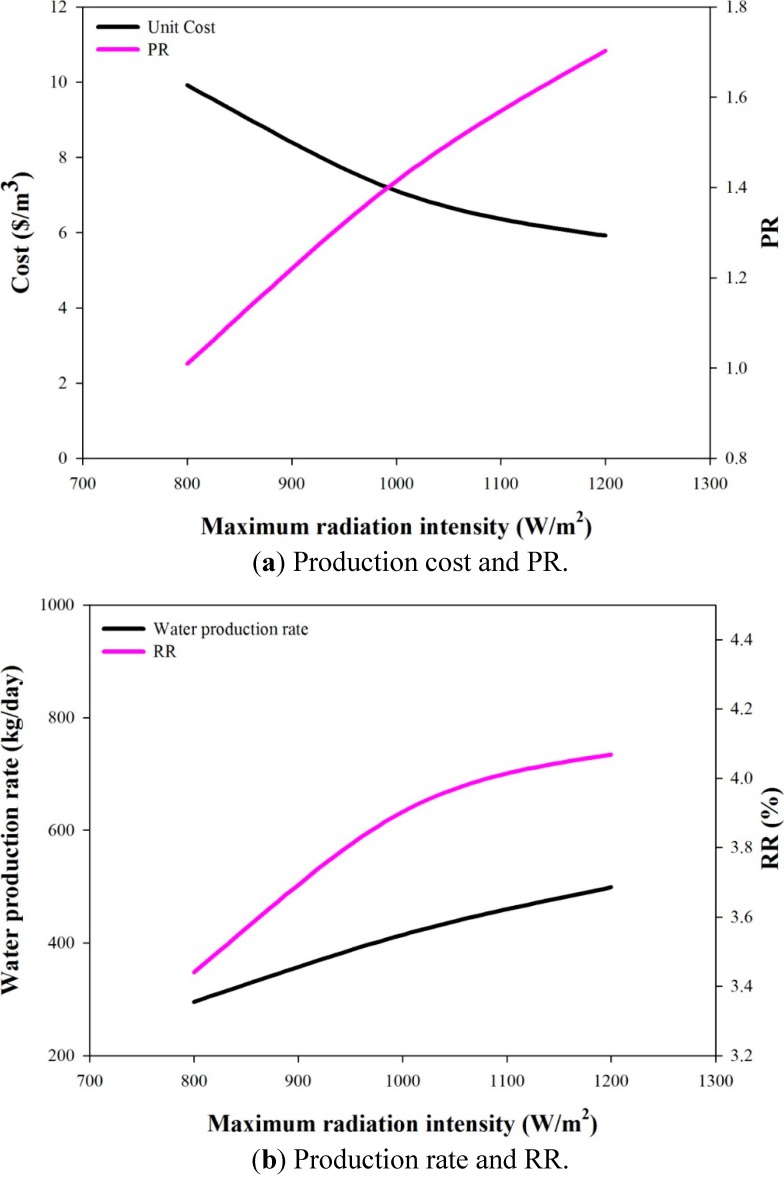
The effects of solar radiation intensity for the AGMD-1-500 kg/day system.

In order to understand how the optimal system designed for high-intensity solar radiation will perform under low-intensity solar radiation conditions, the AGMD-1-500 kg/day system is analyzed for the two solar radiation profiles with lower intensity, as depicted in Figure 4. For each solar radiation profile, the stream flow rates are optimized with the constraints of the maximum capacities of the equipment and with the objective of maximizing the daily water production rate. For the system with fixed equipment sizes, the effects of solar radiation intensity on the unit cost, PR, RR and water production rate are shown in Figure 9a,b. All of these performance indexes decline with the decrease of solar radiation intensity.

### 6.4. Effect of Membrane Characteristics

**Figure 10:**
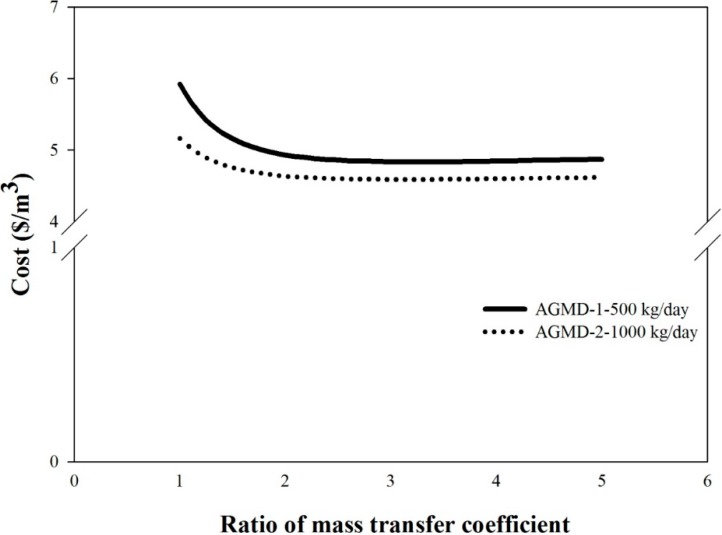
The effect of membrane mass transfer coefficient on unit production cost.

The effect of enhancing membrane characteristics on the overall system performance can be easily investigated by enlarging the mass transfer coefficient of the membrane in the model. The results are shown in Figure 10. For the AGMD-1-500 kg/day system and AGMD-2-1000 kg/day system, double the membrane mass transfer coefficient can result in the reduction of the unit cost by 17% and 11%, respectively. However, a further increase of the membrane mass transfer coefficient cannot provide more cost reduction.

The effect of enhancing the fluid channels of the AGMD modules, such as those reported by Kullab *et al.* [19], on the overall system performance can also be investigated by similar approaches in a future study.

## 7. Conclusions

A systematic method for determining the optimal designs of s-SMDDS that produce water with minimum unit cost has been presented. The method utilizes a pseudo-steady-state approach for equipment sizing and dynamic optimization analysis for taking into account the dynamic nature of the system.

Employing this systematic method, a sound economic evaluation of SMDDS has been performed. For the specified solar radiation profile, the AGMD-1 system that uses an 11.5-m^2^ membrane area should be operated at a 500 kg/day water production rate, and the unit cost with a 1:1 dilution is $5.92/m^3^. On the other hand, the AGMD-2 system that uses a 23-m^2^ membrane area should be operated at a 1000 kg/day water production rate, and the unit cost with a 1:1 dilution is $5.16/m^3^. These costs are about 1/3 of the literature reported data.

For an s-SMDDS system with the given equipment sizes, the system performances, including PR, RR, unit cost and water production rate, are affected by the solar radiation intensity. For the membrane employed in this study, which is a common commercial product, the enhancement of the membrane mass transfer coefficient up to two times can result in the reduction of the unit production cost of the system.

The results obtained from this study are limited to the flat sheet AGMD module with the specified MD sizes and cost functions employed. However, the approaches reported in this paper can be utilized to investigate the optimal design of SMDDS employing different MD configurations, such as LGMD, DCMD and VMD, as well as different membrane modules, such as spiral-wound and hollow fiber.

## Symbol

A: Area (m^2^)
a: Amortization factor
AGMD: Air gap membrane distillation
Cp: Heat capacity (J/kg K)
C: Cost ($ or $/year)
DCMD: Direct contact membrane distillation
D_h_: Hydraulic diameter (m)
D_m_: Molecular diffusivity (m^2^/s)
D_K_: Knudsen diffusivity (m^2^/s)
F: Flow rate (kg/h)
H: Height (m)
h: Heat transfer coefficient (W/m^2^ K)
I: Intensity of solar radiation (W/m^2^)
i: Interest rate
k: Mass transfer coefficient (m/s)
K: Thermal conductivity (W/m K)
L: Length of the equipment (m)
LGMD: Liquid gap membrane distillation
M: Mass of the fluid in the equipment (kg)
MD: Membrane distillation
Mw: Molecular weight of water (kg/kmol)
m: Mass flow rate (kg/s)
N: Mole flux of water (kmol/m^2^ s)
Nu: Nusselt number
n: Plant life (years)
P: Pressure (Pa)
Pr: Prandtl number
Q_h_: Heat flux by convection or conduction (J/m^2^ s)
Q_N_: Heat flux of the sensible heat transfer with the mass flux (J/m^2^ s)
q: Heat transfer rate (J/s)
R: Gas constant (Pa m^3^/kmol K)
Re: Reynolds number
T: Temperature (K)
TAC: Total annual cost ($/year)
t: Time
U: Overall heat transfer coefficient (W/m^2^ K)
V: Volume
VMD: Vacuum membrane distillation
W: Width of the equipment (m)
x: Flow direction
y: Mole fraction

## Greek Letters

ΔH_VL_: Enthalpy for vapor-liquid phase change (J/m^2^ s)
ΔH_vap_: Heat of vaporization (J/kmol)
ΔT: Temperature difference (K)
Δt: Time period (hour)
δ: Thickness (m)
ε: Porosity of the membrane
η: Collector efficiency
τ: Tortuosity of the membrane

## Subscripts

ag: Air gap
air: Air
CC: Capital cost
CL: Cold liquid
cp: Cooling plate
CONL: Condensing liquid
DW: Distilled water
f: Fluid
fixed: Fixed
HL: Hot liquid
HX: Heat exchanger
lf: Liquid film
lm: Logarithmic mean
max: Maximum
MD: Membrane distillation
m1: Hot fluid-membrane interface
m2: Membrane-air gap interface
mem: Membrane
mr: Membrane replacement
O&M: Operating and maintenance
PS: Pseudo state
S: Solar
sat: Saturation
SC: Solar collector
ST: Thermal storage tank
v: Vapor
vap: Vapor
w: Water

## Highlights

- The design and operation of s-SMDDS for minimum-cost water production are obtained.
- A pseudo steady state approach is proposed for equipment sizing.
- Effect of increasing membrane mass transfer coefficient on water production cost is analyzed.
